# EGF-R Protein Expression and Gene Amplification do not Correlate in Pancreas Cancer

**DOI:** 10.4021/gr2008.11.1248

**Published:** 2008-11-20

**Authors:** Gian Luca Baiocchi, Vincenzo Villanacci, Elisa Rossi, Daniela Zanotti, Stefano Maria Giulini

**Affiliations:** a Surgical Clinic, Department of Medical and Surgical Sciences, Brescia University, III Chirurgia, Spedali Civili di Brescia, P.le Spedali Civili, 1, 25123, Brescia, Italy; b Department of Pathology, Brescia University

**Keywords:** pancreas, cancer, EGF-R, FISH, immunohistochemistry

## Abstract

In a series of 13 pancreas cancer specimens, EGF-R was evaluated by means of both immunohistochemistry (IHC) for protein expression and FISH for genetic amplification. The results were discording in 7 cases (IHC positive, FISH negative), while in the remaining 6 cases both IHC and FISH were negative. The possible clinical implications of these results are discussed.

Despite significant advances in systemic therapies, pancreatic cancer remains an often incurable disease; the need for additional therapeutic strategies is hoped to be answered by the way of a better understanding of cellular processes involved in pancreatic cancer. The tyrosine kinase epidermal growth factor receptor (EGF-R) and its ligands are expressed in pancreatic cancer tissues, and this coexpression seems to be correlated with tumour progression [1]. Due to drug costs and to the fear of side effects, it was proposed to select subgroups of patients whose tumours express EGF-R for clinical trial with EGF-R inhibitors [2-4]. In the present work, we investigated the EGF-R expression in 13 patients undergoing pancreatic resection for cancer, including 7 males and 6 females, with middle age 68.2 years; nodal metastases were found in 6 cases, microscopic vascular infiltration in 10 cases and perineural infiltration in 11 cases. The average survival time was 16.9 months (range 10-28 months).

Two different methods of investigation have been used to target the EGF-R: immunohistochemistry (IHC), aimed to evaluate EGF-R protein expression, and fluorescence in situ hybridization (FISH), aimed to evaluate EGF-R gene amplification. IHC was done with the DAKO^®^ kit, employed with little differences from the manufacturer’s recommendations. Slides were deparaffinized and rehydrated in graded solutions of ethanol and distilled water. Endogenous peroxidase was blocked, followed by sequential application of EGF-R primary antibody for 60 minutes and of NovoLink Polymer for 30 minutes (NovoLink Polymer Detection System^®^, Novocastra Laboratories, Newcastle, UK). The immunoprecipitate was visualized by treatment with chromogen for 10 minutes and counterstained by hematoxylin. The expression was evaluated by two observers, following the scoring system suggested by the FDA guidelines (3+, complete and intense membrane staining of >10% tumor cells; 2+, complete but moderate staining of >10% cells; 1+, weak and incomplete staining in >10% cells; 0, no membrane staining, or staining in <10% cells). For the FISH technique, formalin-fixed, paraffin-embedded tissues were cut at 3 mm, mounted on charged slides, and dried. Slides were submitted to enzymatic digestion with 0.005% pepsine (Roche, Germany) for 30 minutes, followed by washing in phosphate buffer saline and fixing by a 10 minutes passage in methanol and acetic acid (3:1 ratio). Fluorochrome-marked DNA probes were employed. The probe for EGFR (Her1/c-erbB; chromosome 7p12) was marked with Spectrum Orange (Vysis Olympus - Milan, Italy), while the centromeric one alpha-satellite, used to look at the centromer of the chromosome 7 (7p11.1-q11.1), was marked with Spectrum Green (Kit PathVysion, Vysis Olympus - Milan, Italy). Target DNA and the probe do pair at the annealing temperature of 37°C overnight in a dark room; after the post-hybridization washing by the detergent NP40 pH 7.00 (Nonidet 40 - Vysis Olympus - Milan, Italy), and coloration by 4,6-diamidino-2-phenylindale (DAPI), the fluorescent microscope (Nikon Optiphot-2) is finally employed for the evaluation. The gene is considered amplified when the ratio between the number of gene signals and its centromer is greater than 2. So, in a cell with a normal number of EGF-R gene copies and of the relative centromer, we will observe respectively 2 orange spots and 2 green spots.

EGF-R protein was expressed by IHC in 7 out of 13 cases, both independent observers concording; in 2 cases the expression was graded +2/+3, in the remaining 5 it was +1. EGF-R gene was amplified in no case. Thus, a mismatch between HIC and FISH was evident from our analysis in almost 7 cases. EGF-R protein expression was evident in 53.8% of cases, and the EGF-R gene was amplified in 0% of cases.

IHC is a technique that, despite its low cost and simple concept, may give divergent results in different series, mainly because of different sensitivity and specificity of the commercially available antibodies, the different systems of tissue processing, the lack of a universal standard of interpretation and the observers’ subjectivity in assessing the results. These problems are confirmed in the present study, as in 8/13 cases a different immunohistochemical score was attributed in the same case by two observers (despite using the same technique and the same microscope). However, this should not explain the absence of correlation between the expression of the protein and the gene amplification in the 7 cases in which the protein was shown to be expressed; a possible explanation may be that other factors, different from the gene amplification, such as genetic mutations or alternative signalling pathways [4], are involved in the receptor activation. This hypothesis introduces some doubts in the field of EGF-R targeted genetic therapy of pancreatic cancer; the reported 0% amplification gene rate seriously questions the more obvious mechanism of EGF involvement in the carcinogenic process.

Further studies are needed to better understand the role of EGF-R in pancreatic cancer. By now, targeted therapies based on EGF-R inhibitors such as Erlotinib should not be decided only on the basis of IHC detection of an over expressed EGF-R.
